# The importance of correct radiological diagnosis in (adenoma malignum): a case report

**DOI:** 10.1093/omcr/omab057

**Published:** 2021-07-21

**Authors:** Elham Shirali, Fariba Yarandi, Narges Izadi Mood, Maryam Rahmani, Marjan Ghaemi

**Affiliations:** 1 Department of Gynecology & Oncology, Yas Hospital, Tehran University of Medical Sciences, Tehran, Iran; 2 Department of Pathology, Yas Hospital, Tehran University of Medical Sciences, Tehran, Iran; 3 Vali-e-Asr Reproductive Health Research Center, Tehran University of Medical Sciences, Tehran, Iran

**Keywords:** minimal deviation adenocarcinoma, adenoma malignum, cervical cancer, magnetic resonance imaging (MRI)

## Abstract

Minimal deviation adenocarcinoma (MDA) of the cervix otherwise known as adenoma malignum is a rare variation of cervical adenocarcinoma. Radiological evaluation plays a great role to ensure an early diagnosis. Here, we report a 48-year-old woman who was presented with a mucoid vaginal discharge 10 years after a supracervical hysterectomy. Despite normal biopsy and cytology, magnetic resonance imaging showed a large cervix and multiple cervical cysts that considered adenoma malignum as a differential diagnosis. She underwent surgery and the pathology confirmed the adenoma malignum. In conclusion, radiologists, as well as gynecologists, and also pathologists may consider MDA among the differential diagnosis in patients with a vaginal discharge and multicysts in the cervix even after hysterectomy despite normal cytology and biopsy.

## INTRODUCTION

Minimal deviation adenocarcinoma (MDA) of the cervix, otherwise known as adenoma malignum, is a rare variety of well-differentiated mucinous adenocarcinoma of the cervix with multiple lobulations of distorted glands [1]. The incidence rate varies between 1 and 3% of all cervical cancers, and its pathology is unrelated to human papillomavirus (HPV) [2].

The major clinical characteristics are mucoid vaginal discharge or bleeding [3]. We report a MDA case which developed after subtotal hysterectomy and was diagnosed with magnetic resonance imaging (MRI) and managed surgically.

## CASE PRESENTATION

A 48-year-old woman (Gravid 2, Para 2) was referred from a private clinic to an academic hospital with a chief complaint of mucoid vaginal discharge for 4 years. She had a history of supracervical hysterectomy 10 years ago due to abnormal vaginal bleeding and uterine myoma. The pathology report showed a large uterus with multiple myoma. In physical examination, cervix appearance was slightly larger than normal and the evidence of chronic cervicitis was observed. The Thin Prep Pap smear was negative for intraepithelial lesions or malignancies. Colposcopy showed chronic inflammation and benign nabothian cysts without any malignant cells. She underwent a vaginal and abdominal ultrasound of cervix that revealed just nabothian cysts.

**Figure 1:**
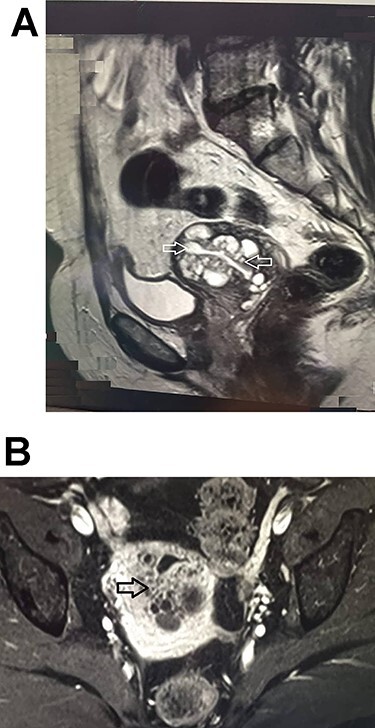
MRI appearance of pelvic before surgery with contrast. There are multiple lesions invading from the endocervical glands to cervical stroma and solid components (**A**). Sagittal T2-weighted image shows the cervical tumor (arrows). (**B**) Axial T1-weighted image of the cervix with irregular multicystic lesions suggesting MDA (arrow).

She performed MRI that showed a large cervix (63 × 50 × 44 mm) and multiple cervical cysts with the different sizes that suggested Tunnel Cluster (Fig. 1A and B). She was on stage Ib with no evidence of adnexal lesion. At this moment, adenoma malignum was considered as a main differential diagnosis that suggested by consultant radiologist.

**Figure 2:**
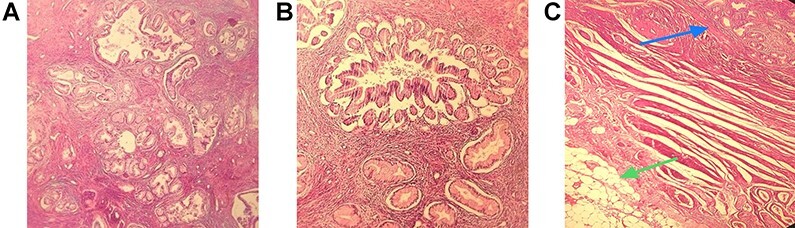
Well-differentiated irregularly shaped, dilated and crowded glands with desmoplastic response (hematoxylin and eosin [H&E] stain) (**A**). Claw or crab-like glands (H&E stain) (**B**). Deeply invasive glands (blue arrow) and pericervical adipose tissue (green arrow) (H&E stain) (**C**).

She underwent laparotomy to remove residues of the uterine and cervix and bilateral salpingo-oophorectomy. Lymph node biopsy was normal. In pathology, the cervix was barrel shaped measuring 3 cm in length and 4 × 4 cm in diameters. The maximal depth of stromal invasion was 14 mm. Microscopic examination reveals atypical architecture including irregularly shaped, dilated and crowded glands with claw or crab-like profiles, intraluminal tufting (Fig. 2A and B), with pale round nuclei with minimal atypia and rare mitoses that invaded deep one-third of cervix (14 mm), near to the pericervical adipose tissue (Fig. 2C) suggesting adenoma malignum.

The upper abdomen imaged was obtained, which was normal. In follow-up after 1 year by MRI and physical examination, she did not have any complication or relapse of the disease.

## DISCUSSION

MDA is a subcategory of mucinous adenocarcinoma of cervix. The mean age at diagnosis is 45 years [4]. Despite its benign appearance in histology, MDA could have an unfavorable prognosis (not necessary poor) due to lymph node metastasis [5], so timely and correct diagnosis is essential. Our case was in stage I and survived after 12 months follow-up without relapse.

Guo *et al*. [1] reported two cases with MDA with watery discharge and enlarged cervix. One case had a history of myomectomy and ovarian cystectomy 10 years before. It may due to the cooperation of adenoma malignum with Peutz–Jeghers syndrome or ovarian tumors [6]. In our case, the pathology of the ovaries was nonspecific and no other abnormalities were detected in upper abdomen.

MDA unlike cervical squamous cell carcinoma is usually located deep within the endocervix. The detection rate of Pap smear, punch biopsy and conization were 7.6, 62.5 and 40% respectively [4]. In our study, the Pap test was negative and the cervical biopsy was normal either, because the mass was very close to the internal os and not easy to access, we preferred a non-invasive diagnostic method with MRI.

A differential diagnosis of adenoma maligna on MRI is pseudoneoplastic glandular lesions and may mimic benign deep endocervical glands. Although normal glands are much fewer in number and have regular spaces, normal architecture and lack of cytological atypia and do not extend beyond 8 mm, but the depth of invasion in our case was 14 mm.

MRI evaluation of our case showed a large cervix and multiple cervical cysts with different sizes that suggested Tunnel Cluster. Multiple cystic lesions are classified based on the volume of solid component, the atypia and cellular activity, and the degree of invasion of the cervical stroma [7]. Lobular endocervical glandular hyperplasias (with or without atypia) are the differential diagnosis, but they have mucoid discharge and packed glands that are uniform without complex morphology and sensible cytological atypia. Indeed, they usually demonstrate no cervical enlargement and no solid component with no stromal invasion [7].

Also, endocervical type adenomyoma is a rare benign tumor similar to MDA and despite a lobular arrangement; it has irregular outlines and papillary infoldings. Other differential diagnosis include HPV-related adenocarcinoma (particularly of mucinous type), endometrial adenocarcinoma and though rare metastatic adenocarcinoma with gastric or pancreatic origin.

HIK-1083 [8], CEA, Ki67 and p53 staining may be useful [9] and P16 as a marker for HPV is typically negative [10]. Considering the differential diagnosis and confirming the diagnosis by pathology and radiology, immunohistochemistry (IHC) reveals overlapping profile with limited value. According to the available evidences, we did not request IHC.

Surgery is the optimal option to manage MDA and including a radical hysterectomy, bilateral pelvic lymphadenectomy and salpingo-oophorectomy at the early stages and for advanced stages chemoradiotherapy is performed [4]. In the cases of hysterectomy with benign indications like ours, subsequent staging operations to define the exact tumor status may be necessary and adjuvant therapy based on surgical outcomes would be indicated. Our patient underwent removal of residual of uterine and cervix with bilateral salpingo-oophorectomy. Lymph node biopsy was negative and no adjuvant therapy was needed.

The prognosis of MDA is known to be relatively poor especially in advanced stages, and it is more aggressive than HPV-associated cervical adenocarcinoma with tendency to lymph node metastasis and early peritoneal and abdominal spread. The 5-year survival is estimated to be 42% compared with 91% for endocervical adenocarcinoma. Otherwise in 1-year follow-up, no sign of further relapse was seen in this case.

## CONCLUSION

In conclusion, timely and prompt diagnosis is very critical in MDA. Therefore, radiologists as well as gynecologists and also the pathologists shall consider MDA among the differential diagnoses in the patients with a vaginal discharge and multicysts in cervix. Future research and longer follow-up are recommended to understand the nature of MDA to provide optimal management.

## Data Availability

The datasets used during the current study are available from the corresponding author on reasonable request.
